# Age-Related Characteristics of Lower-Limb Muscular Balance in Bulgarian Male Football Players Aged 13 to over 19 Years

**DOI:** 10.3390/sports14070306

**Published:** 2026-07-20

**Authors:** Petar Peev, Danail Ivanov, Grigor Gutev

**Affiliations:** National Sports Academy “Vassil Levski”, 1700 Sofia, Bulgaria; petar.peev@nsa.bg (P.P.); gutev@nsa.bg (G.G.)

**Keywords:** strength training, development, injury prevention, isokinetic testing, youth football, bilateral asymmetry, relative strength

## Abstract

Muscle strength and bilateral asymmetry of the lower limbs are relevant to injury risk and performance in football, yet their age-related development in adolescent players outside elite Western European cohorts is insufficiently described. This study determined age-related differences in lower-limb strength and muscular balance in Bulgarian male football players. A total of 122 players aged 13 to over 19 years from three clubs were assessed at the end of the competitive season. Maximal concentric knee extension and flexion peak force of both legs was measured at 60°/s using a system with electronically controlled resistance (Kineo); relative strength (force per body mass) was used for the main between-age comparisons to control for growth. Age differences were analysed with ANOVA and Tukey tests (extension) and Kruskal–Wallis with DSCF comparisons (flexion) across seven age groups. Relative strength differed significantly with age (extension η^2^_p_ = 0.28–0.36; flexion ε^2^ = 0.43–0.49), rising most up to 16 years and then plateauing. The concentric H/Q balance index ranged from 0.48 to 0.62. The findings provide a descriptive developmental profile relevant to individualised strength programming in youth football.

## 1. Introduction

The structure of movements and the nature of football place the players’ locomotor system under enormous strain, and the majority of injuries occur in the lower extremities [1,2,3,4,5,6]. Lower-limb muscle injuries, in particular hamstring strains, are the single most common time-loss injury in professional and youth football and impose a substantial competitive and financial burden on clubs through lost availability, reduced sports form and game rhythm, and diminished player value [7,8,9]. Epidemiological surveillance over consecutive seasons shows that the incidence of these injuries has not declined despite growing investment in prevention, underlining the need to understand their modifiable determinants from an early age [4,6,9,10,11].

Among the intrinsic factors most consistently associated with lower-limb injury are muscle weakness, between-limb strength asymmetry, limited sports experience, and previous trauma or overload [1,2,5,6,12,13,14]. Two indices derived from strength testing are central here. The hamstring-to-quadriceps (H/Q) ratio expresses knee flexor strength as a proportion of knee extensor strength; a lower value indicates a relatively weaker posterior thigh and, conventionally, poorer muscular balance, whereas a value approaching 0.6 indicates more balanced musculature [15,16]. The bilateral (dominant vs. non-dominant) strength difference expresses the asymmetry between limbs; larger values (commonly above 10–15%) have been proposed as indicating elevated injury risk [17,18,19,20]. Recent systematic reviews have, however, questioned the value of the conventional concentric H/Q ratio as an independent predictor of injury, emphasising that it is most informative when monitored longitudinally alongside other factors, and that the functional ratio (eccentric hamstring to concentric quadriceps) is more closely linked to hamstring strain risk during high-speed running [15,21].

In adolescence, these indices are shaped by growth and maturation. Absolute strength rises steeply with body size, so a simple increase in absolute force with age is largely a by-product of growth; relative strength (force per unit body mass) is therefore more informative about genuine neuromuscular adaptation, and the timing of gains depends on biological maturity rather than chronological age alone [22,23,24,25]. Isokinetic strength and muscular balance in youth football have been characterised mainly in elite Western and Central European academies [26,27], where players often follow highly structured, professionally staffed development programmes. Whether comparable developmental patterns hold in national contexts with different training structures, such as Bulgaria, has not been established; documenting them is a first step toward judging whether reference values derived from Western cohorts are transferable. We make no claim of anatomical or physiological differences between Eastern and Western European players; rather, differences in training volume, professionalisation and playing calendar may plausibly influence the developmental profile, and a descriptive baseline for Bulgarian players is currently lacking.

We hypothesised that relative lower-limb strength would increase non-linearly across age groups, with the most pronounced gains during peak adolescence, while the H/Q ratio and bilateral asymmetry would fluctuate in an age-specific manner before stabilising. This study aimed to determine age-related differences in lower-limb muscle strength in Bulgarian male football players, and specifically (i) to describe the strength of the knee extensors and flexors and (ii) to describe the bilateral muscular asymmetry of the lower extremities across seven age groups from 13 to over 19 years.

## 2. Materials and Methods

### 2.1. Participants

The study sample comprised 122 male football players aged 13 to over 19 years with complete strength measurements. Players were recruited from three football clubs in Bulgaria. Inclusion criteria were male sex, current systematic participation in football training and competition, and being free of injury at testing. Exclusion criteria were any lower-limb musculoskeletal injury within the two months before testing and any incomplete strength assessment; on this basis, five players from the original cohort of 127 were excluded for missing strength data, leaving 122. Because the 19-year category contained only five players, it was merged with the over-19 (senior) category to form a single “19 years and over” group (n = 30) for all analyses, giving seven age groups; this avoids unstable estimates and low statistical power in a single small cell. Participant characteristics are presented in Table 1.

The weekly training load differed by age in a graded manner typical of academy football. All training sessions lasted approximately 90 min. Players aged 13–14 years trained four times per week plus one match, and players aged 15 years and older trained five times per week plus one match. Training in all groups was predominantly football-specific; systematic external-load resistance training was not a formal, individually periodised component of the programme during the study period. This graded increase in football exposure with age should be considered when comparing the present data with cohorts following more heavily resistance-based development programmes.

Testing was conducted at the end of the competitive period over 10 days, between 14:00 and 18:00. Age and the dominant (preferred kicking) leg were registered before testing; the dominant leg was defined as the leg self-selected to kick a ball for maximal distance. The study followed the Declaration of Helsinki and was approved by the University Human Research Ethics Committee of the National Sports Academy “Vassil Levski”, Sofia (No. 1456/06.04.26). Written informed consent was obtained from all adult participants and from a parent or legal guardian for participants under 18 years, and the clubs gave written permission to record and analyse the data.

### 2.2. Anthropometry

Body mass was recorded with an InBody 230 analyser (InBody Co., Ltd., Seoul, Republic of Korea) and stature with a Seca 213 stadiometer (SECA GmbH & Co. KG, Hamburg, Germany). Body mass was measured in the morning, in football shorts and without shoes, in kilograms; stature was measured barefoot to 0.1 cm.

### 2.3. Strength Assessment

Muscle strength was assessed with a training system with electronically controlled resistance (Kineo Intelligent Load System, Technogym S.p.A., Cesena, Italy). Testing was performed in the manufacturer’s isokinetic concentric mode with the movement speed preset to 60°/s; the device supports preset angular velocities between 12 and 778°/s, and the manufacturer describes the isokinetic method as movement at a constant speed irrespective of the force applied. The system is cable- and pad-driven: only the ankle pad was adjusted to each participant, and individual tibial (lever-arm) lengths were neither measured nor entered as study variables. The device reports the peak linear force at the contact pad, which we express in kilograms-force (kgf); it also reports angular quantities (peak position and range of motion in degrees), but it does not output joint torque (N·m), and the manufacturer does not publicly document the internal transformation between cable displacement and knee angular velocity. Consequently, the reported strength values are peak linear pad forces, not peak torques, and we could not convert them to N·m; absolute magnitudes are therefore not directly comparable with those obtained from rotary isokinetic dynamometers. Devices integrating a load cell into a motor-driven resistance unit have shown excellent test–retest reliability and high concurrent validity against criterion isokinetic dynamometry for lower-limb strength [28,29]. All measurements were concentric.

Testing was carried out by a trained team of four sports scientists (two UEFA A-licence football coaches and two strength-and-conditioning specialists holding a doctoral degree), all instructed in and following the same written, standardised testing protocol; the same protocol, warm-up, positioning and instructions were applied to every player across the three clubs to maximise consistency. Formal intra- and inter-tester reliability coefficients (e.g., ICC, typical error) were not computed because repeated (test–retest) measurements were not performed; this is acknowledged as a limitation.

Peak force was measured for the quadriceps and hamstring musculature of both legs. Relative strength was calculated as peak force divided by body mass (kgf·kg^−1^) and was used as the primary variable for between-age comparisons, so that differences reflect neuromuscular adaptation rather than growth in body size; absolute values are additionally reported for completeness and comparison with the literature. Bilateral asymmetry was expressed, per movement, as the absolute dominant–non-dominant difference divided by the larger value × 100. The knee muscle balance index was the concentric H/Q ratio (peak flexion force/peak extension force) per leg, averaged across legs; higher values indicate more balanced knee musculature and lower values a relatively weaker hamstring. Each athlete performed three repetitions per leg (30 s rest between legs); all performed extension first, then flexion after 5 min rest, and the highest of the three repetitions was retained.

### 2.4. Procedures

Players completed a standardised warm-up (4 min cycle-ergometer at 90 rpm, 4 min general exercise and stretching, 10 squats and 10 lunges). Leg extension was performed seated and belted to minimise hip contribution, with the pad at the ankle; leg curl was performed standing, the pelvis on a cushioned plate and the pad on the distal third of the calf, pushing with the hamstrings only.

### 2.5. Statistical Analysis

Analyses used SPSS (v27.0; IBM Corp., Armonk, NY, USA) and jamovi (v2.6). Normality was assessed with the Shapiro–Wilk test and homogeneity of variance with the Levene test. Between-age differences in relative strength were tested with one-way ANOVA (Welch where variances were unequal) and Tukey post hoc tests for normally distributed variables (extension), and with the Kruskal–Wallis test and Dwass–Steel–Critchlow–Fligner (DSCF) post hoc comparisons for non-normally distributed variables (flexion). Effect sizes were partial eta-squared (η^2^_p_) for ANOVA and epsilon-squared (ε^2^) for Kruskal–Wallis. One player lacked a body-mass value and was therefore excluded from relative-strength analyses (n = 121 for those analyses; n = 122 for absolute values). Significance was set at *p* < 0.05. A sensitivity/power analysis for a seven-group one-way ANOVA (α = 0.05) indicated that, at the smallest observed effect on relative strength (partial η^2^ = 0.28, Cohen’s f = 0.62), the achieved power with the present sample (N = 121) exceeded 0.99, and that effects as small as partial η^2^ ≈ 0.11 were detectable at 80% power; the sample was therefore adequately powered for the reported between-age comparisons.

## 3. Results

**Figure 1:**
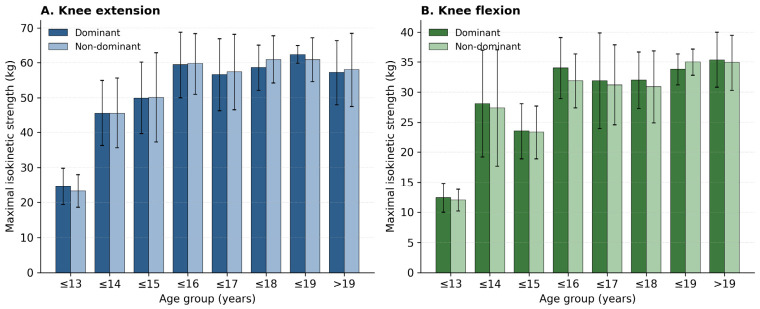
Relative isokinetic strength (mean ± SD; peak force per unit body mass) of the dominant and non-dominant legs in (**A**) knee extension and (**B**) knee flexion across age groups.

Relative knee strength by age group is shown in Figure 1 and Table 2, with absolute values in Table 3. Relative extension strength increased from 0.62 kgf·kg^−1^ (dominant) at 13 years to a peak of 0.89–0.90 kgf·kg^−1^ at 16 years, then declined slightly to 0.76 kgf·kg^−1^ in the 19-and-over group; relative flexion strength followed a similar pattern, peaking at 16 years (0.48–0.51 kgf·kg^−1^). Thus, once body mass was accounted for, strength did not increase monotonically with age but rose to a mid-adolescent peak and then stabilised or slightly decreased.

**Figure 2:**
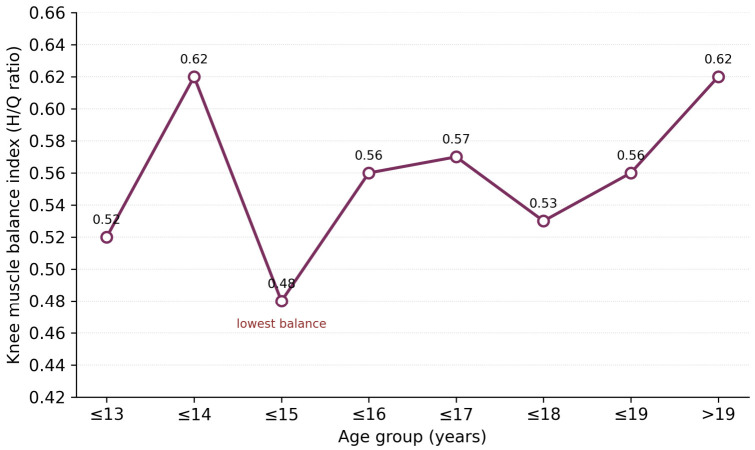
Knee muscle balance index (concentric hamstring-to-quadriceps ratio) across age groups. Higher values indicate more balanced knee musculature.

Bilateral asymmetry and the knee muscle balance index are summarised in Table 4. Extension asymmetry was largest in the youngest (13 years, 11.7%) and the senior (19-and-over, 10.1%) groups and smaller (4.3–8.9%) in between; flexion asymmetry ranged from 5.4% to 10.4%. The concentric H/Q balance index (Figure 2) ranged from 0.48 to 0.62, being lowest at 15 years and highest in the 14-year and senior groups.

Relative strength differed significantly across age groups for all four measures. For extension, one-way ANOVA showed a significant, moderate-to-large effect of age (dominant: F(6, 114) = 7.40, *p* < 0.001, η^2^_p_ = 0.28; non-dominant: F(6, 114) = 10.79, *p* < 0.001, η^2^_p_ = 0.36). Tukey post hoc tests showed that 13-year-olds had significantly lower relative extension strength than all older groups (*p* < 0.05), with the 16-year group also differing from the 14-year and 19-and-over groups; the remaining older groups did not differ significantly. For flexion, the Kruskal–Wallis test showed a significant, large effect of age (dominant: H(6) = 61.73, *p* < 0.001, ε^2^ = 0.49; non-dominant: H(6) = 54.51, *p* < 0.001, ε^2^ = 0.43), with 13-year-olds again significantly weaker than older groups (*p* < 0.05). No significant differences were found among the 15-and-older groups for either movement (*p* > 0.05).

## 4. Discussion

The main finding is that, once body mass is taken into account, lower-limb strength in these Bulgarian male football players did not increase monotonically with age but rose to a peak around 16 years and then plateaued, with the clearest difference separating 13-year-olds from all older groups. Analysing relative rather than absolute strength is important: absolute force nearly doubles between the youngest and oldest groups (Table 3), but this largely reflects the increase in body mass from ≈40 kg at 13 years to ≈78 kg in the senior group, i.e., growth rather than a genuine change in neuromuscular capacity. Expressing strength per unit body mass removes most of this size effect and reveals the underlying developmental pattern [22,25,30].

This pattern is consistent with the biology of maturation. Strength gains in youth are governed by biological maturity rather than chronological age, and the fastest gains cluster around and shortly after peak height velocity, typically in early-to-mid adolescence [23,24,31,32,33,34]. The steep rise up to 16 years plausibly reflects this maturational window—increases in muscle cross-sectional area and pennation angle, tendon stiffness, motor-unit recruitment and neural drive that accompany puberty—whereas the subsequent plateau in relative strength suggests that, after mid-adolescence, further absolute gains are largely proportional to continued growth in body mass rather than disproportionate neuromuscular improvement. The graded training load in this academy (three to five sessions per week by age) may also contribute, but the largely football-specific, non-periodised strength stimulus is unlikely by itself to explain the mid-adolescent peak. We interpret these mechanisms cautiously, as maturity status, muscle architecture and tendon properties were not directly measured.

Direct comparison with Western and Central European cohorts is instructive. Maly et al. [26], studying elite Czech youth players with rotary isokinetic dynamometry, likewise reported that between-age differences in body-mass-normalised knee strength diminish in later adolescence, and Bishop et al. [27], in professional and under-18 English players, found asymmetry values broadly similar to ours. Fousekis et al. [35] reported greater asymmetry in players with less experience, matching the larger asymmetry we observed in our youngest group. The convergence of our normalised values and asymmetry ranges with these cohorts suggests that, despite a different training structure, the developmental profile of Bulgarian academy players is broadly comparable, and that reference ranges derived from Western European players are, tentatively, applicable. Direct numerical comparison of absolute magnitudes is limited by our use of linear pad force (kgf) rather than joint torque (N·m).

In most groups, the non-dominant leg produced greater or equal relative extension force, which we attribute to the stabilising role of the supporting leg in sport-specific actions; this is supported by other work [26,35,36]. Bilateral asymmetry approached or exceeded 10–11% mainly in the youngest (13 years) and senior groups, indicating limited specific adaptation in the youngest players and a re-emergence of asymmetry among seniors, consistent with the idea that greater asymmetry accompanies both immaturity and, later, position-specific specialisation [20,35,37,38].

The concentric H/Q balance index (0.48–0.62) is in the range reported for male soccer players at low angular velocities [16]. However, our clinical interpretation is deliberately cautious. We measured only the conventional concentric H/Q ratio, which provides a general picture of muscular balance but has limited independent predictive value for hamstring strain injury; systematic reviews conclude that the conventional ratio is not, on its own, a reliable injury predictor, and that the functional ratio (eccentric hamstring to concentric quadriceps) and a documented eccentric-strength deficit are more closely related to strains sustained during high-speed sprinting [15,21,39,40,41]. Our H/Q values should therefore be read as a descriptive index of muscular balance rather than as a screening threshold for injury risk; we do not claim that the players with lower concentric ratios are necessarily at higher risk of hamstring strain. Establishing risk would require eccentric and functional H/Q assessment and prospective injury surveillance.

The hamstrings are critical during running and stability tasks [42,43,44,45,46,47], and hamstring training should combine hip- and knee-dominant exercises for elite football [48,49], reflecting the eccentric demand of the late swing phase of sprinting [50]. From a practical standpoint, the mid-adolescent window of fastest relative-strength gain (up to ≈16 years) is when structured, progressive strength work may be most productive; the graded football load documented here provides a realistic backdrop for such programming. The elevated asymmetry in the youngest and senior groups highlights these as priority targets for individualised, symmetry-oriented work. Because the conventional H/Q ratio is only a weak standalone indicator, balance and asymmetry data are best tracked longitudinally rather than used as one-off cut-offs [15,16].

### Limitations and Future Research

This study has several limitations. Participants came from only three Bulgarian clubs, and the sample, though adequate, was unevenly distributed across age groups; even after merging the 19-year group, some cells remained modest, limiting the precision of comparisons at the upper age range. The cross-sectional design precludes inferences about individual trajectories. Strength was recorded as linear pad force (kgf) rather than joint torque (N·m), which constrains direct comparison of absolute magnitudes with rotary-dynamometer studies. Only concentric strength was measured; the Kineo system also permits eccentric and concentric/eccentric assessment, and eccentric hamstring strength and the functional H/Q ratio are the more relevant metrics for hamstring-injury risk, representing the logical next step. Maturity status, muscle architecture and tendon properties were not measured, so mechanistic explanations remain tentative. Finally, formal test–retest reliability (ICC, typical error) was not established because repeated measurements were not performed; although a trained team applied a single standardised protocol, future work should quantify reliability directly. In particular, sitting height was not collected, so a somatic maturity offset could not be computed and players could not be classified as pre- or post-peak-height-velocity (PHV) using established prediction equations [24]; incorporating a maturity-offset assessment and a pre-/post-PHV analysis is an important priority for future studies, which would allow the developmental pattern reported here to be interpreted in explicitly maturational terms.

## 5. Conclusions

In these Bulgarian male football players, relative lower-limb strength increased most up to about 16 years of age and then plateaued, with 13-year-olds clearly weaker than all older groups; the apparent continued rise in absolute strength largely reflects growth in body mass. Bilateral asymmetry approached the 10–11% range mainly in the youngest and senior groups, and the concentric H/Q balance index (0.48–0.62) was comparable to values reported for Western European cohorts. The developmental profile provides a descriptive baseline for Bulgarian youth football and supports individualised strength programming; because only the concentric H/Q ratio was assessed, its use for injury-risk screening should be cautious, and eccentric/functional assessment is recommended for future work.

## Figures and Tables

**Table 1:** Sample size, height and body mass (mean ± SD) of the athletes by age group.

Age Group	n	Height (cm)	Body Mass (kg)
Up to 13 years	16	156.3 ± 8.6	39.8 ± 4.3
Up to 14 years	19	161.6 ± 9.4	60.4 ± 10.7
Up to 15 years	19	173.1 ± 8.5	62.6 ± 11.1
Up to 16 years	17	175.5 ± 9.7	66.6 ± 7.8
Up to 17 years	11	177.0 ± 4.7	70.9 ± 6.9
Up to 18 years	10	179.4 ± 5.8	71.9 ± 5.6
19 years and over	30	181.3 ± 6.3	77.0 ± 7.1

Values are mean ± SD. Stature and body mass were measured individually for every participant (Seca 213 stadiometer; InBody 230).

**Table 2:** Relative isokinetic strength (kgf·kg^−1^ body mass; mean ± SD) at 60°/s by age group.

Age Group	Ext. Dom.	Ext. Non-Dom.	Flex. Dom.	Flex. Non-Dom.
Up to 13	0.62 ± 0.13	0.59 ± 0.11	0.31 ± 0.06	0.30 ± 0.05
Up to 14	0.77 ± 0.15	0.76 ± 0.12	0.47 ± 0.14	0.46 ± 0.15
Up to 15	0.80 ± 0.10	0.80 ± 0.10	0.38 ± 0.04	0.37 ± 0.04
Up to 16	0.89 ± 0.11	0.90 ± 0.12	0.51 ± 0.06	0.48 ± 0.04
Up to 17	0.80 ± 0.10	0.81 ± 0.10	0.45 ± 0.09	0.44 ± 0.07
Up to 18	0.82 ± 0.08	0.85 ± 0.09	0.45 ± 0.07	0.43 ± 0.09
19 and over	0.76 ± 0.13	0.76 ± 0.14	0.46 ± 0.05	0.46 ± 0.07

Ext., extension; Flex., flexion; Dom., dominant leg. Values in kilograms-force per kilogram body mass. n = 121 (one player lacked body mass).

**Table 3:** Absolute isokinetic peak force (kgf; mean ± SD) at 60°/s by age group, provided for comparison with the literature.

Age Group	Ext. Dom.	Ext. Non-Dom.	Flex. Dom.	Flex. Non-Dom.
Up to 13	24.6 ± 5.2	23.3 ± 4.7	12.4 ± 2.4	12.0 ± 1.8
Up to 14	45.6 ± 9.3	45.6 ± 10.0	28.1 ± 8.9	27.4 ± 9.7
Up to 15	49.9 ± 10.2	50.1 ± 12.8	23.5 ± 4.6	23.3 ± 4.4
Up to 16	59.4 ± 9.4	59.7 ± 8.7	34.0 ± 5.1	31.9 ± 4.5
Up to 17	56.6 ± 10.3	57.4 ± 10.8	31.9 ± 8.0	31.2 ± 6.7
Up to 18	58.6 ± 6.5	61.0 ± 6.8	32.0 ± 4.7	30.9 ± 6.0
19 and over	58.0 ± 8.6	58.5 ± 9.9	35.1 ± 4.3	34.9 ± 4.3

Values are peak linear force at the contact pad (kgf), not joint torque. n = 122.

**Table 4:** Bilateral asymmetry (%) and concentric H/Q balance index (mean ± SD) by age group. Both variables are computed directly from raw peak forces and therefore include all 122 players (n = 122; the participant lacking a body-mass value is excluded only from the relative-strength analyses in Table 2).

Age Group	Ext. Asymmetry (%)	Flex. Asymmetry (%)	H/Q Index
Up to 13	11.7 ± 9.2	7.3 ± 5.8	0.52 ± 0.09
Up to 14	8.9 ± 7.9	10.4 ± 8.3	0.62 ± 0.20
Up to 15	8.3 ± 7.7	8.8 ± 6.0	0.48 ± 0.06
Up to 16	7.9 ± 6.3	7.3 ± 5.1	0.56 ± 0.06
Up to 17	4.3 ± 3.2	7.0 ± 4.2	0.57 ± 0.15
Up to 18	6.6 ± 4.9	9.3 ± 4.4	0.53 ± 0.07
19 and over	10.1 ± 10.5	6.9 ± 5.6	0.61 ± 0.09

## Data Availability

The original contributions presented in this study are included in the article/supplementary material. Further inquiries can be directed to the corresponding author.

## Supplementary Materials

The following supporting information can be downloaded at https://www.mdpi.com/article/10.3390/sports14070306/s1, S1 Dataset (raw individual data for 122 players, including relative strength and H/Q ratios).
